# Bone Density, Osteocalcin and Deoxypyridinoline for Early Detection of Osteoporosis in Obese Children

**DOI:** 10.3889/oamjms.2015.092

**Published:** 2015-09-07

**Authors:** Ghada El-Dorry, Hala Ashry, Tarek Ibrahim, Tahany Elias, Fatma Alzaree

**Affiliations:** 1 *Institute of Postgraduate Childhood Studies, Ain Shams University, Medical Studies Department, Cairo, Egypt*; 2 *National Research Center, Child Health Department, Cairo, Egypt*; 3 *National Research Center, Medical Biochemistry Department, Cairo, Egypt*

**Keywords:** DEXA parameters, osteocalcin, calcium, phosphorus, alkaline phosphatise, obese Egyptian children

## Abstract

**AIM:**

This study aimed at comparing between bone density using DEXA, serum osteocalcin and urinary DPD in obese and non obese prepubertal children.

**METHODS:**

After taking the consent of eighty children they were subjected to: full examination, anthropometric measurements, blood samples were withdrawn to determine serum osteocalcin, Ca, Ph, alkaline phosphatase, and urinary DPD. Bone densities, body composition of the whole body were examined using DEXA. Data were analyzed using SPSS.

**RESULTS:**

All anthropometric variables showed significant increase in obese children except for height in comparison to control group. Total mass, lean + BMC, lean, fat, area, BMC, BMD and Z score of the whole body were significantly increased in obese children. Serum calcium showed significant increase while alkaline phosphatase was significantly decreased in obese children. DPD showed no significant difference between obese and non obese children. Significant negative correlation was found between ca, lean, lean + BMC and total mass. Serum alkaline phosphatase showed also a significant negative correlation with (lean + BMC and total mass). Serum osteocalcin showed negative significant correlation with area, BMC, BMD, lean and Z score.

**CONCLUSION:**

Obese children showed significant increase in anthropometric and DEXA parameters, increase in serum calcium and significant decrease in serum alkaline phosphatase. Osteocalcin was negatively correlated with most of DEXA results.

## Introduction

World health organization (WHO) has defined obesity as abnormal or excessive fat accumulation that may impair health [1].

Obesity also can be defined as a condition of excess fat that results when excess energy has been accumulated, and is associated with a large number of life threatening disorders. It occurs when energy intake exceeds energy expenditure. In other words, it is an abnormal growth of adipose tissue due to an enlargement of the fat cell size [2].

Overweight and obesity are the fifth leading risk for global deaths. At least 2.8 million adults die each year as a result of being overweight or obese. In addition, 44% of the diabetes burden, 23% of the ischemic heart disease burden and between 7% and 41% of certain cancer burdens are attributable to overweight and obesity. Body mass index (BMI) is a simple index of weight-for-height that is commonly used to classify overweight and obesity in adults. It is defined as a person’s weight in kilograms divided by the square of his height in meters (kg/m^2^). WHO has defined BMI greater than or equal to 25 as overweight and BMI greater than or equal to 30 as obesity [1].

Osteoporosis is a major health problem; it is a disease of progressive bone loss associated with increased risk of fractures. The disease often develops unnoticed over many years, with mild symptoms and signs, until fractures occur [3]. It develops as a result of imbalance between bone resorption and bone formation [4]. Although it is considered as a disease of the elderly, there is now universal agreement that osteoporosis has pediatric origin because if individuals fail to achieve optimal peak bone mass (PBM) and strength in childhood and adolescence, there is a likelihood of development osteoporosis later life [5]. Awareness of osteoporosis and its complications is growing; so, there is increasing reasons to develop strategies for screening in order to target treatment more effectively and reduce the number of fractures [3].

During the past 10 years, DEXA has emerged as a cost-effective, safe and accurate means to quantitate skeletal mass. The WHO has adopted DEXA derived bone mineral density (BMD) measurements to define normal bone, osteopenia and osteoporosis [1]. Bone status can be described by measuring BMD, which provides information on both bone mineral content and bone fragility. Bone mineral density measurement, however, does not provide data on the rate of bone remodeling. This information is obtained, qualitatively, by measuring biochemical bone markers. Both measurements complement each other and are needed to get a clear understanding of bone status. Many physiological and pathological processes may influence bone metabolism resulting in changes in serum concentration of bone turnover markers. Measurements of these parameters offer many advantages for investigating skeletal diseases in children and adolescents as well as monitoring the response to treatment [6].

Osteocalcin, the major non-collagenous protein, synthesized by osteoblasts plays an important role in the regulation of bone growth and in the correct deposition of the minerals in the matrix. Its expression follows the proliferative phase of osteoplastic differentiation, so it can be considered a marker of mature osteoblasts. Serum levels of Osteocalcin and deoxypyridinoline are not stable throughout our life and are greater in infants and children than in adults. Peak values occur at puberty [6, 7].

The bone resorption marker deoxypyridinoline (DPD) reflects the level of osteoclastic activity in the bone-remodeling process. Even when BMD is not in the osteoporotic range, increases in urine DPD indicate increased osteoclastic-bone resorption and risk for fracture. Therefore, the spread of obesity among prepubertal children and frequent occurrence of fractures raises the need for a tool for early detection of osteoporosis among obese children [8].

The aim of the present study was to compare between bone density using DEXA and serum Osteocalcin level (as a bone formation marker) beside urinary DPD level (as a bone resorption marker) in obese and non-obese prepubertal children for early detection of osteoporosis in childhood.

## Subjects and Methods

This case-control study included eighty (80) Egyptian children, aged from 6 to10 years, both males and females subdivided into 40 cases (obese) and 40 controls (non-obese). They were enrolled from the outpatient clinic of the Institute of Postgraduate Childhood Studies, Ain Shams University during the period from September 2012 till March 2013, with simple exogenous obesity; whose BMI exceeding 95^th^ percentile for obese and above 5^th^ to less than 85^th^ percentile for non-obese according to the Egyptian Growth Charts [9]. Children with Congenital and endocrinal causes of obesity or under treatment with corticosteroid therapy were excluded from the study. Written informed consent was taken from all patients’ parents before enrollment in the study and after full explanation of their role in the research. The consent was approved by the ethical committee of the National Research Center and Institute of postgraduate childhood studies, Ain Shams University.

### Methods

All children included in the study were subjected to the following:

1. *Full history taking*.
2. *Thorough clinical examination*.

- General examination: pulse, blood pressure and temperature.

- Systemic examination: head and neck, cardiac, chest, abdominal and neurological examination.

3. Anthropometric measurements and auxology

All anthropometric measurements were obtained using standardized equipment and following the recommendations of the International Biological program [10]: body weight, body height, BMI: Calculation according to the known formula: BMI = weight (kg)/height (m^2^).

4. Laboratory Investigations

One fasting blood sample will be collected in the first visit; (5 cc venous blood samples). Samples will be collected in plain vacutainers and subsequently, serum will be separated and stored at -20°C until assay will be performed. While urine is collected in a sterile container, centrifugation for 20 minutes at the speed of 2000-3000 r.p.m., remove supernatant. If precipitation appeared, centrifuge again. The assay includes: serum Osteocalcin, serum calcium, serum inorganic phosphate, serum alkaline phosphatise, and urinary deoxypyridinoline (DPD).

5. Bone mineral density assessment

A real bone mineral density BMD (g/cm^2^) of the whole body and the whole body composition parameters (whole body bone mineral content (g), lean body mass (g), whole body fat (g) and fat %) were done to each subject at the Institute of Postgraduate Childhood Researches, Ain Shams University. All of the parameters mentioned above were taken using the Hologic QDR Discovery DEXA fan-beam scanner (software v. 12.1, fast-array mode). Calibration stability was monitored using two site- specific phantoms (Hologic Anthropomorphic Spine and Whole Body phantoms) that are scanned weekly. All scans were analyzed using Hologic software release 12.3. This software release has special features for pediatric scans. The whole body analyses use an automatic low bone density detection algorithm that increases the sensitivity of finding low density bone [11].

*Precautions done before DEXA scanning:* - Artefacts, including enteric tubes, metallic objects and jewellery, were excluded from the image; - We had to give sedatives to some children in order to ensure child stability all over the scanning time especially whole body scan (takes long duration) [12].

6. Statistical analysis

Data were analyzed using standard computer program SPSS Windows version (17) (SPSS Corporation, USA). Descriptions of all data were performed as quantitative data and were presented as mean, SD (standard deviation), median and range. The correlation coefficient (r) was used to interrelate the parametric data, and Chi- square test to differentiate between percentages cases and controls.

After analysis a probability p value: P > 0.05 was considered insignificant; P < 0.05 was considered significant; and P < 0.01, <0.001 and < 0.0001were considered highly significant.

## Results

**Figure 1:**
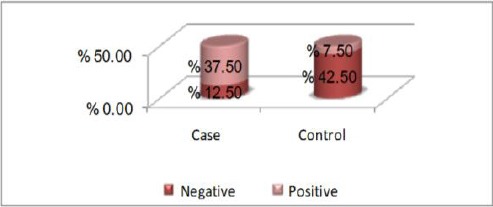
*Distribution of the studied sample according to family history of obesity*.

Figure 1 shows distribution of the sample according to family history of obesity where we found that family history is negative in 42.5% out of 50% in controls, while it was positively related in 37.5% out of 50%in cases.

**Figure 2:**
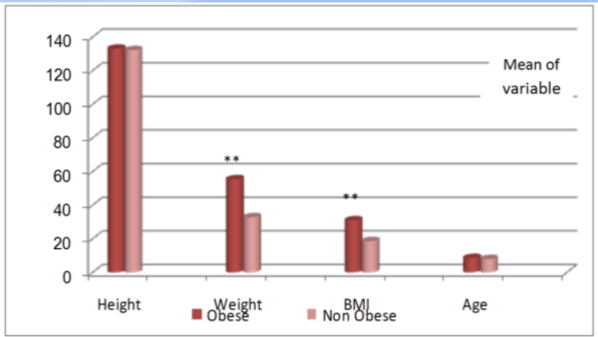
Age and anthropometric measurements of the studied sample. **, highly significant at p value <0.01; BMI, body mass index.

Figure 2 shows age and anthropometric measurements of the studied sample where all variables showed significant increase in obese children in comparison to control group except for the height.

**Figure 3:**
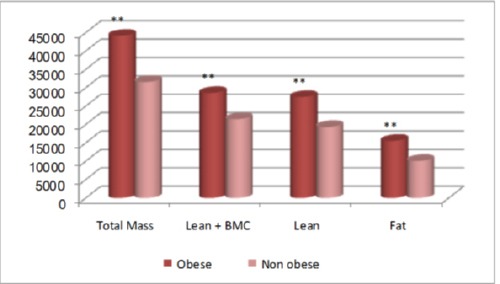
Comparison between obese and non-obese children concerning total mass (g), lean+BMC (g), lean (g) and fat (g) in DEXA results for the whole body. **, p<0.01 highly significant.

**Figure 4:**
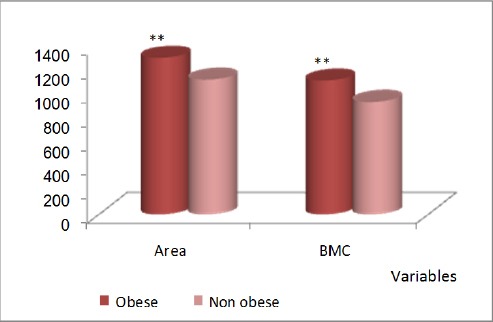
Comparison between obese and non-obese children concerning area (cm^2^) and BMC (g) in DEXA results for the whole body. **, p<0.01 highly significant; BMC, bone mineral content.

**Figure 5:**
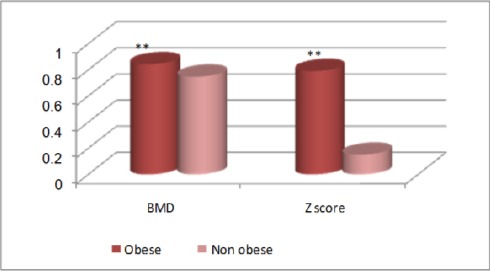
Comparison between obese and non-obese children concerning BMD (g/cm^2^) and Z- score in DEXA results for the whole body. **, p<0.01 highly significant; BMD, bone mineral density.

Figures 3, 4, and 5 show comparisons between obese and non-obese children in DEXA results for the whole body, where all DEXA parameters for the whole body showed significant increase in obese children more than the non-obese children.

**Figure 6:**
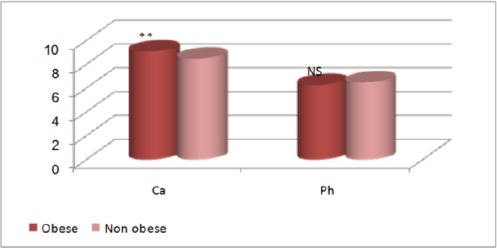
Comparison between obese and non-obese children in calcium and phosphorus. **, p<0.01 highly significant; NS, non- significant; Ca, Calcium; Ph, phosphorus.

**Figure 7:**
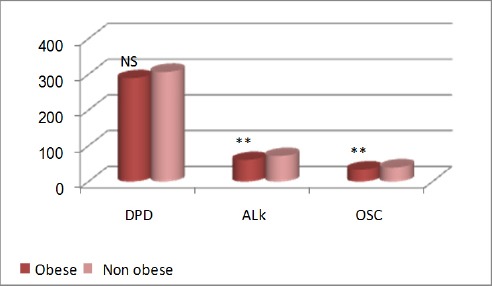
Comparison between obese and non-obese children in laboratory parameters (DPD-lipid profile-Alkaline phosphatase-OC). **, p<0.01 highly significant; NS, non –significant.

Table 1 and Figures 6 and 7 show comparison between obese and non-obese in laboratory variables; where calcium showed significant increase while alkaline phosphatase showed significant decrease in obese children. Osteocalcin showed significant decrease in obese children, while DPD showed no significant difference between obese and non-obese children.

**Table 1:** Comparison between obese and non-obese in laboratory parameters

	Non obese N = 40		Obese N = 40		t test	P value
	Mean ± SD	Range	Mean ± SD	Range		
Ph	6.49±0.67	5.47-7.60	6.27±0.69	4.93-7.47	1.48	>0.05
OSC	39.06±7.29	23.27-51.20	33.13±11.83	10.60-58.15	2.69	<0.01**
DPD	306.55±194.81	86.12-652.58	289.02±130.17	112.86-602.27	0.473	>0.05
Ca	8.48±0.73	6.79-9.32	9.08±0.72	7.96-10.60	3.75	<0.01**
ALk-ph	70.88±16.67	36.16-94.43	59.98±11.67	23.90-75.58	3.39	<0.01**

** p<0.01highly significant

NS, non- significant; DPD, deoxypyridinoline; Alk.ph, alkaline phosphatise; Ca, Calcium Ph: phosphorus.

## Discussion

Childhood and adolescence are important phases of the human development during which the adult bone mass density is determined and therefore, problems during this period of life could compromise bone health in adulthood.

Our all anthropometric parameters are increased (except for height) in obese children. this results goes with that of Cobayashi et al., [13] and Abou El-Soud et al., [14], which stated that anthropometric measurements are good indicators of obesity in children.

Also our results goes in accordance with Elkhayat et al., [15], who showed significantly higher mean values of weight, weight for age, BMI, waist, hip and mid upper arm circumferences, waist: hip ratio as well as biceps, triceps, subscapular, suprailiac and abdominal skin folds thickness among cases than controls. Meanwhile, Salem et al., [16], recommended that BMI should be used routinely to screen for overweight and obesity in children and adolescents as it is easy to use in a clinical setting and correlates with subcutaneous and total body fat.

**Table 2:** Correlation between DEXA parameters for the whole body and Calcium, Phosphorus, Alkaline phosphatase, Osteocalcin & deoxypyridinolin

DEXA Parameter	Ca (mg/dl)		Ph (mg/dl)		Alk.ph. (µ/L)		OSC (ng/ml)		DPD (nmol/l)	
	r	p	r	p	r	p	R	p	r	p
Area (cm^2^)	0.051	>0.05	0.057	>0.05	0.070	>0.05	**-0.228**	**<0.05**	0.022	>0.05
BMC(g)	0.115	>0.05	0.217	>0.05	0.094	>0.05	**-0.357**	**<0.01**	0.068	>0.05
BMD (g/cm^2^)	0.109	>0.05	0.168	>0.05	0.118	>0.05	**-0.411**	**<0.01**	0.048	>0.05
Fat(g)	0.004	>0.05	0.160	>0.05	0.214	>0.05	0.030	>0.05	0.020	>0.05
Lean(g)	0.096	>0.05	**-0.246**	**<0.05**	0.135	>0.05	**-0.238**	**<0.05**	0.078	>0.05
Lean + BMC (g)	0.100	>0.05	**-0.297**	**<0.01**	**-0.232**	**<0.05**	0.209	>0.05	0.078	>0.05
Total Mass	0.052	>0.05	**-0.246**	**<0.05**	**-0.238**	**<0.05**	0.097	>0.05	0.032	>0.05
Z score	0.032	>0.05	0.115	>0.05	0.098	>0.05	**-0.292**	**<0.01**	0.130	>0.05

* p<0.05 significant

** p<0.01highly significant

P>0.05 non-significant, r, correlation coefficients.

The fact that anthropometric measurements are accepted sensitive indicators of growth patterns, obesity and health status of a child as stated by Chatterjee et al., [17], and the positive relation between obesity and body composition especially body fat previously shown by Cobayashi et al., [13], and Abou El-Soud et al., [14] goes in accordance with the results of the present study.

Regarding bone parameters, our results showed that, (BMD, BMC, area, fat, lean, lean + BMC, total mass and z-score) for the whole body, hip and lumbar areas are greater in obese children, which confirm the relationship between them and obesity. All of bone measurements are done and gave the same results. So we can perform either of them (i.e. measuring the whole body alone or with hip or lumbar spine).

Koyama et al., [18] reported that obese children had significantly greater fat as well as lean compartments, both total and regional and also they had higher total BMC and regional BMC values than normal - weight children. Also, Clark et al., [19] reported that, fat mass is positively related to bone area, in view of the strong positive relationship between bone size and both BMD and BMC.

Ellis [20], Hasanoglu et al., [21], and Cobayashi et al., [13] found that the median prevalence of bone mineral density is twice as frequent among post pubertal adolescents who suffer from obesity and overweight (69.3%) than among their normal weight counterparts (32.1%).

In contrast with our findings, other investigators have reported no difference between obese and non-obese stated by Manzoni et al., [22] or decreased BMD and BMC in obese children reported by Goulding et al., [23].

There are several possible mechanisms for increased bone mass in childhood obesity. Hormonal influences such as increased circulating leptin concentrations. Leptin acts as a growth factor on the chondrocytes of skeletal growth centers via insulin-like growth factor-1 receptor expression and thereby potentially contributes to the increased linear growth and skeletal mass observed in childhood obesity stated by Maor et al., [24].

In addition, to increased conversions of rostenedione to estrogen may play a role. As it is known that, during puberty estrogen promotes accrual of bone mass on the cortical endosteal surface and in trabecular bone. Also, both androgens and estrogens stimulate calcium absorption and retention and result in a net positive flow of calcium into bone, which contribute to bone accumulation reported by Schoenau et al., [25], and Thomas & Burguera [26].

Increased biomechanical loading due to increased body weight and increased lean muscle mechanical forces may also have contributed to the increased bone dimensions and mass observed in the obese subjects. Increased loading of long bones produces the greatest mechanical stresses on the sub-periosteal surface and stimulates bone formation by sub-periosteal expansion stated by Frost [27]. Also, the increase in BMD in relation to increased BMI measured by DEXA was explained by Leonardo et al., [28] on the basis that DEXA is a two-dimensional approach that provides an estimate of density in (gm /cm^3^) which shows only little information about real bone strength.

We propose that the increased BMC in childhood obesity results in increased bone strength but this increase is not sufficient to overcome the significantly greater forces generated when an obese child falls on an outstretched arm. This can be explained by the increased biochemical loading due to increased body weight and increased lean muscle mechanical forces that may also have contributed to the increased bone dimensions and mass observed in the obese subjects.

Although childhood obesity may result in an increased risk of childhood forearm fractures, the effect of obesity on life-long fracture risk is unknown.

Our results showed that, all DEXA parameters (BMD, BMC, area, fat, lean, lean + BMC, total mass and z-score) for the whole body were positively correlated with BMI, weight and height.

Junior et al., [29] reported a positive relationship between body weight and BMD. Indeed, obese subjects, from an early age, have an increased bone density mainly due to the stress occasioned by the increased weight on bone tissue that causes deformation and hence leads to bone remodelling. This was previously shown by Do Pardo et al., [30], 2009 and Rhie et al., [31].

On the contrary, Zamboni et al., [32] evaluated the bone mineralization of obese children and stated that, obese children had reduced mineral density in comparison with normal-weight peers.

Also, Goulding et al., [23] found that overweight and obese children have lower bone area and bone mass relative to body weight than their leaner peers.

Cao [33] reported that the decreased bone mass with obesity may be due to increased marrow adipogenesis at the expense of osteoblastogenesis and / or increased osteoclastogenesis because of up regulated production of pro inflammatory cytokines and/or reduced adiponectin production and / or reduced calcium absorption associated with high fat intake.

Ellis [20], Leonardo et al., [28], and Nagasaki et al., [34] stated that body weight might improve bone mineralization in obese children by increasing the mechanical loading on weight-bearing bones. Also, Rocher et al., [35] stated that in obese children, the skeleton must be stronger than in controls to support their higher body weight. However, the bone mass ratio to total body weight was significantly lower in obese children in comparison to controls (2.46% ± 0.46% vs. 3.53% ± 0.46%, p < 0.0001), suggesting that the skeleton is not sufficiently resistant to support the higher body weight of obese people.

In this study we measured some important laboratory parameters for bone status as calcium, phosphorus and alkaline phosphatase in addition to serum Osteocalcin and urinary DPD. Both bone markers that predict the bone status; were not compared to the results of body composition and bone parameters obtained by DEXA in any previous researches.

Serum calcium level in our study showed significant increase in obese children. This increase in serum calcium was previously shown by Zemel et al., [36] who found that there is significant increase in serum calcium of obese children.

This can be explained by the excess intake of all food supplements by the obese children in this age, so it will give us a false interpretation about the strength of their bones, as most of us will think that they have strong bones although their bones are not as strong as it is expected and this increase is in the volume only without real increase in strength.

As for alkaline phosphatase our study showed significant decrease in obese children. This decrease in serum alkaline phosphatase that was found in our obese children also showed a strong negative correlation with lean+ BMC, while Marwaha and Sripathy [37] found that, among the common bone mineral parameters, only serum alkaline phosphatase had a significant negative association with BMD. In another study by Pettifor and Moodley [38], it was shown that serum alkaline phosphatase has a negative correlation with BMD and subjects with alkaline phosphatase >300 IU had lower BMD than those with normal levels. So our results concerning the decreased alkaline phosphatase goes in accordance with these studies but the strong negative correlation differs from a study to another.

Osteocalcin which is the bone formation marker chosen in our study found that its total serum levels were significantly lower in obese children. This goes in accordance with Wang et al., [39], who found the same result in overweight and obese children.

Again, it was found that osteocalcin is negatively correlated with area, BMC, BMD, lean mass and z-score, measured for whole body, hip and lumbar spine in obese children in comparison to non-obese children. Similar results were reported Wang et al., [39], where they found that serum osteocalcin level was negatively correlated with fat percentage and visceral fat area. These findings indicate that body composition is negatively related to osteocalcin levels in obese children.

Another study by Bonofiglio et al., [40] conducted in adolescent girls had documented that serum osteocalcin was negatively correlated with BMD at ultra-distal and proximal radius of the forearm.

It is known that, the bone resorption marker (deoxypyridinoline DPD) reflects the level of osteoclastic activity in the bone-remodelling process and a higher risk for osteoporotic hip fracture, independent of BMD. Even when BMD is not in the osteoporotic range, increases in urine DPD indicate increased osteoclastic-bone resorption and risk for fracture as stated by McCormic et al., [8].

In the present study, the urinary DPD which was used as bone turnover marker showed no significant difference between obese and non-obese groups. So, we can use osteocalcin as an early predictor of osteoporosis in obese children to avoid continuation of the problem of osteoporosis in the adult period especially in pre and post-menopausal females. While for the urinary DPD, as it did not give us any significant data either in obese or non-obese children in addition to its high price, so we do not recommend the use of the urinary DPD as a bone turn over marker in this early age.

In conclusion, obese children have increased anthropometric and DEXA parameters which were positively correlated with BMI, weight, height and lipid profile except for HDL. Obese children also showed significant increase in serum calcium and significant decrease in alkaline phosphatase. Osteocalcin was found to be negatively correlated with most of DEXA results in obese children in comparison to non-obese children. While the urinary DPD, showed no significant difference between obese and non-obese groups.

Recommendations: Preventive measures should be implemented quite early so that obesity and its co morbidities can be controlled through participation in physical activities and through an adequate diet.

## References

[1] WHO: Waist Circumference and Waist-Hip Ratio, Report of a WHO Expert Consultation 8–11 December (2008).

[2] Kiess W, Petzold S, Topler M, Garten A, Bluher S, Kapellen T (2008). Adipocyte and adipose tissue. Best Pract Res Clin Endocrinal Metab.

[3] Scott AC, Tonnarelli B, Papadimitropoulos A, Scherberich A (2010). Recapitulation of endochondral bone formation using human adult mesenchymal stem cells as a paradigm for developmental engineering. PNAS.

[4] Naim MM, Khashayar S (2006). Treatment of osteoporosis in patients with chronic liver disease and in transplant recipients. Current treatment options in Gastroenterology.

[5] SubLim J (2010). Pediatric dual energy X-ray absorptiometry: Interpretation and clinical and research application. Korean J of Pediatrics.

[6] Ambroszkiewicz J, Klemarczyk W, Gajewska J, Chełchowska M, Laskowska-Klita T (2007). Serum concentration of biochemical bone turnover markers in vegetarian children. Adv Med Sci.

[7] Yang L, Ali AM, Saleh M, Olongaro S (2006). Experimental model of tibial plateau fracture for biomechanical testing. J Biomech.

[8] McCormick B, Stone I, corporate analytical team (2007). Economic costs of obesity and the case for government intervention. Obesity reviews.

[9] Ghalli I, Salah N, Hussien F, Erfan M, El-Ruby M, Mazen I, IntSartorio A, Buckler JMH, Marazzi N (2008). Egyptian growth curves 2002 for infants, children and adolescents.

[10] Hiernaux J, Tanner JM, Weiner J.S., Lourie S.A. (1969). Growth and physical studies. Human Biology: A guide to field methods.

[11] Kalkwarf HJ, Zemel BS, Gilsanz V (2007). The bone mineral density in childhood sudy: Bone mineral content and density according to age, sex, and race. J ClinEndocrnol and Metab.

[12] Binkovitz LA, Henwood MJ (2007). Pediatric DEXA technique and interpretation. Pediatr Radiol.

[13] Cobayashi F, Lopes LA, Taddei JA (2005). Bone mineral density in overweight and obese adolescents. J Pediatr (Rio J).

[14] Abou El-Soud NH, Youssef MM, Mohsen MA, Kazem YA (2006). Obesity in children and adolescents: Effect on bone mineral content and density. J Med Sci.

[15] El-Khayat HA, Emam EK, Hassan NE, Kandeel WA, Elagouza IA, Zaki ME (2013). Impact of body fat mass on bone mineral density and content and on serum level of c-terminal telopeptide of type 1 collagen among overweight and obese children and adolescents. Journal of Applied Sciences Researches.

[16] Salem MA, El Alfy MS, El Beblawy NM, Tash F, Zaki M, Farid S (2002). Prevalence of obesity in school children and its link to type 2 diabetes mellitus. Egyptian Journal of Pediatrics.

[17] Chatterjee S, Chatterjee P, Bandyopadhyay A (2006). Skinfold thickness, body fat percentage and body mass index in obese and non-obese Indian boys. Asia Pacific J Clin Nut.

[18] Koyama H, Nishizaura Y, Yamshita M (1990). Measurement of composition changes using dual photon absorptiometry in obese patients undergoing semi starvation. Metabolism.

[19] Clark EM, Ness AR, Tobias JH, the Avon Longitudinal Study of Parents and Children Study Team (2006). Adipose tissue stimulates bone growth in prepubertal children. J Clin Endocrinol Metab.

[20] Ellis KJ (2000). Human body composition: in vivo methods. Physiological Review.

[21] Hasanoglu A, Bideci A, Cinaz P, Tumer L, Unal S (2000). Bone mineral density in childhood obesity. J Pediatr Endocrinol Metab.

[22] Manzoni P, Brambilla P, Pietrobelli A (1996). Influence of body composition on bone mineral content in children and adolescents. Am J Clin Nutr.

[23] Goulding A, Taylor RW, Jones IE (2000). Overweight and obese children have low bone mass and area for their weight. Int J Obes Relat Metab Disord.

[24] Maor G, Rochwerger M, Segev Y, Phillip M (2002). Leptin acts as a growth factor on the chondrocytes of skeletal growth centers. J Bone Miner Res.

[25] Schoenau E, Neu CM, Rauch F, Manz F (2001). The development of bone strength at the proximal radius during childhood and adolescence. J Clin Endocrinol Metab.

[26] Thomas T, Burguera B (2002). Is leptin the link between fat and bone mass?. J Bone Miner Res.

[27] Frost HM (1987). The mechanostat: a proposed pathogenic mechanism of osteoporosis and the bone mass effects of mechanical and nonmechanical agents. Bone Miner.

[28] Leonardo MB, Shults J, Wilson BA, Andrew M, Tershakovec AM, Zemel BS (2004). Obesity during childhood and adolescence augments bone mass and bone dimensions. Am J Clin Nutr.

[29] Júnior IF, Cardoso JR, Christofaro DG, Codogno JS (2013). The relationship between visceral fat thicknesses and bone mineral density in sedentary obese children and adolescents. BMC Pediatrics.

[30] Do Prado WL De, Piano A, Lazaretti-Castro M, De Mello MT, Stella SG, Tufik S (2009). Relationship between bone mineral density, leptin and insulin concentration in Brazilian obese adolescents. J Bone Miner Metab.

[31] Rhie YJ, Lee KH, Chung SC, Kim HS, Kim DH (2010). Effects of body composition, leptin, and adiponectin on bone mineral density in prepubertal girls. J Korean Med Sci.

[32] Zamboni G, Soffiati M, Givarina D, Tato L (1998). Mineral metabolism in obese children. Acta Pediatrica.

[33] Cao JJ (2011). Effects of obesity on bone metabolism. Journal of Orthopedic Surgery and Research.

[34] Nagasaki K, Kikuchi T, Hiura M, Uchiyama M (2004). Obese Japanese children have low bone mineral density after puberty. J Bone Miner Metab.

[35] Rocher E, Chappard C, Jaffre C, Benhamou C, Courteix D (2008). Bone mineral density in prepubertal obese and control children: relation to body weight, lean mass, and fat mass. J Bone Miner Metab.

[36] Zemel MB, Thompson W, Zemel P (2002). Dietary calcium and dairy products accelerate weight and fat loss during energy restriction in obese adults. Am J Clin Nutr.

[37] Marwaha RK, Sripathy G (2008). Vitamin D & bone mineral density of healthy school children in northern India. Indian J Med Res.

[38] Pettifor JM, Moodley GP (1997). Appendicular bone mass in children with a high prevalence of low dietary calcium intakes. J Bone Miner Res.

[39] Wang JW, Tang QY, Ruan HJ, Caiw W (2013). Relationship between serum osteocalcin levels and body composition in obese children. J Pediatr Gastroentrol Nutr.

[40] Bonofiglio D, Maggiolini M, Catalano S, Marsico S, Aquila S, Giorno A (2000). Parathyroid hormone is elevated but bone markers and density are normal in young female subjects who consume inadequate dietary calcium. Br J Nutr.

